# Multifunctional Nanocarpets for Cancer Theranostics: Remotely Controlled Graphene Nanoheaters for Thermo-Chemosensitisation and Magnetic Resonance Imaging

**DOI:** 10.1038/srep20543

**Published:** 2016-02-04

**Authors:** Arathyram Ramachandra Kurup Sasikala, Reju George Thomas, Afeesh Rajan Unnithan, Balasubramaniam Saravanakumar, Yong Yeon Jeong, Chan Hee Park, Cheol Sang Kim

**Affiliations:** 1 Department of Bionanosystem Engineering, Graduate School, Chonbuk National University, Jeonju 561-756, Republic of Korea; 2 Division of Mechanical Design Engineering, Chonbuk National University, Jeonju 561-756, Republic of Korea; 3 Department of Radiology, Chonnam National University Hwasun Hospital, Chonnam National University Medical School, Gwangju 501-746, Republic of Korea; 4 Department of Mechatronics Engineering, Jeju National University, Jeju, 690-756, Republic of Korea

## Abstract

A new paradigm in cancer theranostics is enabled by safe multifunctional nanoplatform that can be applied for therapeutic functions together with imaging capabilities. Herein, we develop a multifunctional nanocomposite consisting of Graphene Oxide–Iron Oxide -Doxorubicin (GO-IO-DOX) as a theranostic cancer platform. The smart magnetic nanoplatform acts both as a hyperthermic agent that delivers heat when an alternating magnetic field is applied and a chemotherapeutic agent in a cancer environment by providing a pH-dependent drug release to administer a synergistic anticancer treatment with an enhanced T_2_ contrast for MRI. The novel GO-IO-DOX nanocomposites were tested *in vitro* and were observed to exhibit an enhanced tumoricidal effect through both hyperthermia and cancer cell-specific DOX release along with an excellent MRI performance, enabling a versatile theranostic platform for cancer. Moreover the localized antitumor effects of GO-IO-DOX increased substantially as a result of the drug sensitization through repeated application of hyperthermia.

Nanotechnology is rapidly advancing, and there is a growing interest towards the field of nanomedicine. The unique properties of nanotechnology-based therapeutics have attracted interest of scientists from various research areas, especially in the field of cancer theranostics^1^. The frontiers of cancer research are currently focusing on developing methods for early detection of cancer and on finding cancer treatments that have few side effects^2^. To this end, biocompatible magnetic nanoparticles (MNPs) play an important role in cancer theranostics because they offer a combination of properties, including magnetic resonance imaging (MRI), cell and tissue targeting, drug delivery and hyperthermia^3^,^4^,^5^. Recently, there has been a critical interest towards achieving synergistic cancer theranostics by combining the unique heat generation property of magnetic nanoparticles in an alternating magnetic field (AMF) to induce hyperthermia, the unique drug delivery properties that are specific against cancer cells, and the improved ability of the magnetic nanoparticles to accelerate the MRI relaxation process as T2 contrast agents for MRI^6^,^7^,^8^,^9^.

Hyperthermia therapy with super paramagnetic iron oxide nanoparticles (SPIONs) is widely recognized for its safety and effectiveness in treating cancer relative to conventional forms of therapy^10^. The nanoparticles participate in cancer drug delivery through controlled, targeted delivery of therapeutics at tumor sites^11^,^12^. The therapeutic drugs are conjugated to these nanoparticles through several mechanisms, including surface absorption^13^, hydrogen bonding^14^, chemical conjugation^15^, and so on. However, the main disadvantage of nanoscale drug carriers that have been developed for combined hyperthermia and chemotherapy is the low drug loading capacity due to their restricted size and slim surface coating, thus limiting the particle surface area^16^. It is therefore critical to improve the loading efficiency of the drug in order to provide effective anticancer functionality. Thus, biologists have expressed an increased interest to develop highly efficient drug delivery platforms along with hyperthermia.

Graphene oxide (GO) and its derivatives have made a profound impact on materials science due to their potential use in a wide range of applications. Over the past few years, there seems to be an augmented interest in applying GO and its derivatives for various biomedical ends, include photodynamic therapy, gene/drug delivery, molecular imaging, and so on^17^. The use of GO and its derivatives has gaining popularity in these research areas for numerous reasons, including their high biocompatibility, excellent electrochemical and optical properties, and a capability to adsorb a variety of aromatic biomolecules through a π-π stacking interaction and/or electrostatic interaction. Moreover, the abundant oxygen-containing groups in GO provide an excellent platform for further modifications using a stimuli responsive polymers or targeting ligands to facilitate targeted imaging and smart drug delivery^18^,^19^.

Recently, some studies have demonstrated the potential use of superparamagnetic nanoparticles incorporated with graphene oxide (GO) to form magnetic composites intended for use in various biomedical applications^20^,^21^. The main advantage of using these magnetic composites in hyperthermia-based treatment is the synergistic improvement in hyperthermic properties due to the high thermal conductivity of GO^22^. Here, we explore the possibilities of using a versatile Graphene Oxide–Iron Oxide-Doxorubicin nanocomposite (GO-IO-DOX) as a theranostic cancer platform that combines hyperthermia, chemotherapy, and MRI. The GO-IO-DOX was prepared with the goal of developing a new material that will greatly contribute to the current research of highly complex diseases, such as cancer. The overall concept of the present study is shown in Fig. 1. Thus the prepared GO-IO-DOX exhibit a synergistic anticancer effect from both their hyperthermic response in an AMF and cancer cell-specific DOX release. Moreover a very good MRI performance can also be realized with GO-IO-DOX, proving its efficiency as a versatile theranostic platform for cancer treatment.

## Results and Discussion

The biocompatible Graphene oxide-Iron oxide nanocomposites (PEGylated GO-IO nanocomposite (GO-IO-PEG)) were prepared using a simple, versatile strategy that was reported earlier by Peng *et al*^23^. The unique drug loaded multifunctional nanoplatform for synergistic cancer theranostics has been synthesized by encapsulating an anticancer drug doxorubicin onto the GO-IO-PEG by simple mixing to initiate physisorption^24^. The as-obtained GO-IO-DOX material was investigated in terms of its potential theranostic applicability for cancer that combines hyperthermia, chemotherapy and MRI. A detailed explanation of the synthesis of the magnetic nanoparticles and graphene oxide as well as the procedure followed to prepare GO-IO-DOX is given in the supporting information and the schematic diagram (Figure S1) also illustrates the same.

**Figure 2:**
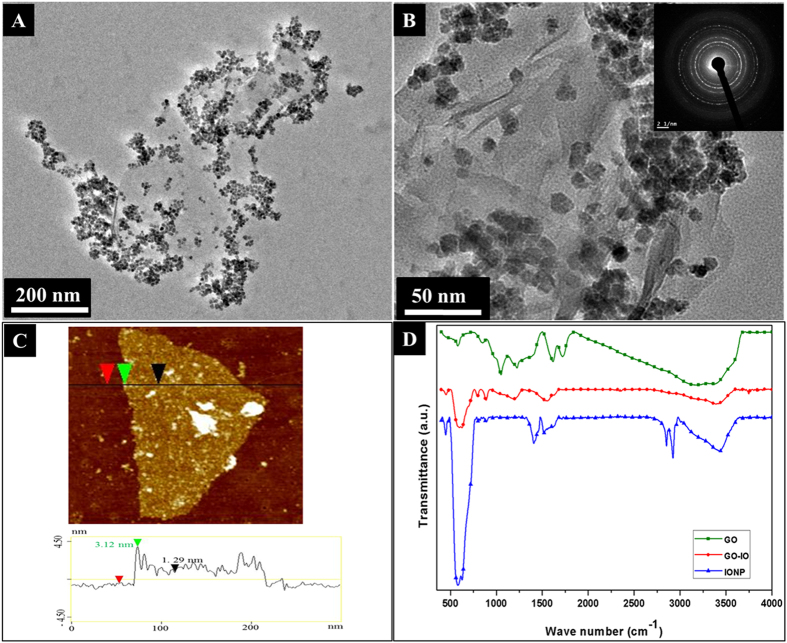
(A) TEM images of the GO-IO nanocomposite, (**B**) TEM image at a higher magnification of the GO-IO nanocomposite with an inset showing the SAED patterns, (**C**) AFM image of the GO-IO nanocomposite, (D) FTIR spectra of IONP, GO and GO-IO nanocomposite.

Synthesis of magnetic nanoparticles (IONPs) is carried out using high-temperature thermal decomposition method^25^. The amount of surfactants and solvents is varied to obtain monodisperse IONPs with a size smaller than 20 nm. The size and morphology of the as-prepared Fe_3_O_4_ nanoparticles and the GO (modified Hummer’s method^26^) were inspected using high-resolution transmission electron microscopy (HRTEM) and the corresponding SAED image (Figure S2 of the supporting information). The structural features and morphology of the GO-IO nanocomposites were studied by X-ray diffraction XRD patterns (supporting information) and HRTEM. The XRD patterns indicating the peak positions and the relative intensities of IONPs, GO and the GO-IO nanocomposites are presented (in Figure S3 of the supporting information). All of the diffraction peaks that were present in the XRD spectrum of the as-prepared IONPs were in good agreement with the standard diffraction card JCPDS 19-062. The diffraction peaks present in the GO-IO nanocomposites exhibited characteristic peaks for both the IONPs and GO, thereby confirming their presence with a high crystallinity in the nanocomposite. The TEM images (Fig. 2A,B) clearly indicate a uniform distribution of the IONPs on the surface of the GO sheets without any apparent changes in size or morphology. The SAED pattern (inset of Fig. 2B) also specifies the existence of IONPs on the surface of the GO without any damage to the crystal structure, especially after the formation of the GO-IO nanocomposites. The morphology of the GO and the GO-IO nanocomposite was further investigated using AFM (Fig. 2C). The cross section analysis of AFM proves that GO used as the carriers of IONPs possess a height of ~1 nm, suggesting a single layer graphene sheet^27^. Furthermore, many round surface protuberances with size 3–4 nm height are observed on the surface of the GO–IO nanocomposite, which characterizes for IONPs with a very few aggregation^28^.

FTIR was performed over the spectral range from 500 to 4000 cm^−1^ to further examine the bonding between the GO and IONPs and the presence of the different functional groups on the GO–IO nanocomposites (Fig. 2D). The characteristic peak of the IONPs can be determined as the strong absorption at 580 cm^−1^ for both IONPs and GO-IO peaks, and this band corresponds to the vibrations of the Fe–O group, which indicate the presence of bonds between the Fe_3_O_4_ nanoparticles^29^. Moreover FTIR confirms the reaction between the amine-reactive epoxy group and the amine functional group from oleylamine due to the reduction in the epoxide stretching vibrations at 1120–1250 cm^−1^ of GO–IO. Hence, amide covalent bonding formed, as indicated by the successive appearance of the –C–N– stretching vibrations of amides at 1456.3 cm^−1^ along with the overlap in the C=O stretching vibrations and amide –N–H bending vibrations at 1610.5 cm^−1^. The non-covalent bonding addition of oleylamine to GO through COO−NH_3_^ + ^ionic bonding was proven by the vanishing of the –C=O (from –COOH) stretching vibration at 1705 cm^−1^ and the appearance of two new peaks at 1610.5 and 1456.3 cm^−1^ corresponding to the asymmetric and symmetric stretching vibrations of –COO−. Additionally the presence of characteristic oleylamine peaks at 2910.6 and 2850.8 cm^−1^ corresponds to the symmetric and asymmetric stretching vibrations of –CH_2_. The =CH_2_ bending vibrations at 937.2 cm^−1^ and –CH_2_ bending vibrations at 709.8 cm^−1^ were confirmed in the FTIR spectra^23^. Raman spectroscopy was also carried out to further confirm the presence of graphene oxide and iron oxides with different structural phases (Figure S4 of the supporting information) and found that GO–IO nanocomposite shows typical magnetite peaks, and the characteristic GO peaks at around 1324 cm^−1^ and 1592 cm^−1^ correspond to the D and G bands, respectively are present in the Raman spectrum^30^ confirming the presence of both Fe_3_O_4_ and GO with in the GO-IO nanocomposite.

**Figure 3:**
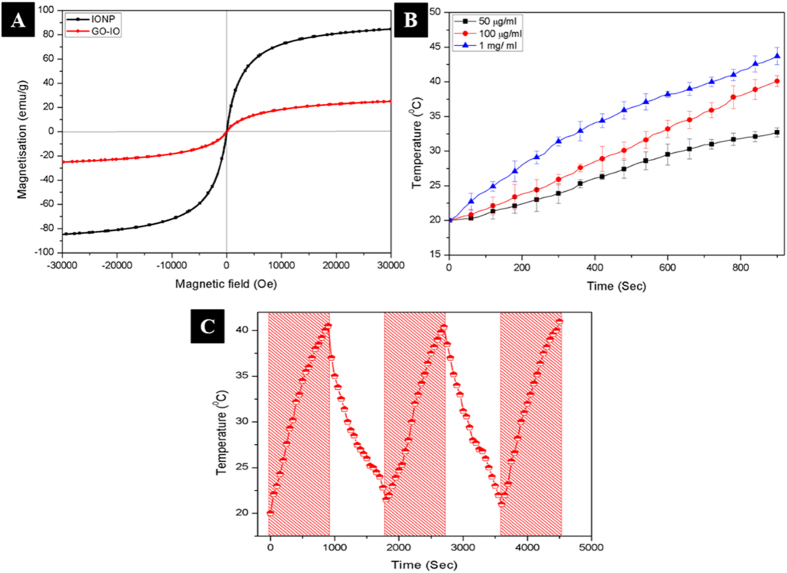
(**A**) Magnetic hysteresis curves of the IONP and GO-IO nanocomposites, (**B**) Magnetic field dependent heating ability of GO-IO-PEG nanocomposites at various concentrations, (**C**) Cyclic heating profile of GO-IO-PEG nanocomposites (the shaded zones indicate that the magnetic field has been ON)

Magnetic properties of the as prepared IONPs and GO-IO nanocomposites were analyzed by measuring the magnetization as a function of the applied magnetic field at 300K (Fig. 3A). The hysteresis loops clearly indicate that both samples retained their superparamagnetic properties with no coercivity or remanence. The IONPs and GO-IO nanocomposites displayed a saturation magnetization of 84.95 emu/g and 25.37 emu/g, respectively, and the decreased saturation magnetization of the GO-IO nanocomposites can be explained through the results of the thermal gravimetric analysis (TGA) (Figure S5 of the supporting information). The TGA result of the GO-IO nanocomposites reveals that GO-IO nanocomposites endures a weight loss of ~70.05% when heated to 800 °C due to the removal of organic moieties. Therefore, the remaining 29.95% of the magnetic components from the GO-IO nanocomposites can be attributed to the saturation magnetization. Moreover, the Ms values that were estimated by using the TGA data^31^ are in good agreement with the Ms values that were obtained for the GO-IO sample by using the physical property measurement system. The magnetic field-dependent heating ability and the cyclic heating profile of GO-IO-PEG nanocomposites was measured for various IONP concentrations in the GO-IO-PEG nanocomposites, and the samples obtained at a 100 μg/ml concentration were found to be suitable for hyperthermia application with a SAR value of 557.38 W/g (Fig. 3B,C). GO was selected as the base platform for IO nanoparticles due to its excellent thermal conductivity and in case of polymer coated or hydrogel coated IO nanoparticles^8^,^32^,^33^, the presence of such encapsulation or covering can act as the heat barrier, which will hinder the efficient heat release to the surrounding environment. In our case, the excellent thermal conductivity of GO sheets has been contributed towards the enhanced conductivity of the composite material and thereby GO-IO composite exhibited higher heating ability and efficient hyperthermia performance to heat up the cancer tissue surroundings very effectively without any insulating barriers in between.

The pH-sensitive release of DOX from GO-IO-DOX was assessed by testing the release of DOX in buffer solutions at pH values of 5 and 7.4 to mimic the pH of a tumor site and of normal tissue or blood, respectively. The characteristic UV–vis absorbance peak indicates the DOX loading of the GO–IO–DOX sample (Figure S8 of the Supporting Information). The drug release studies clearly indicate that DOX exhibited a pH-dependent release from GO-IO-DOX (Figure S9 of Supporting Information) with a loading efficiency of 95.8% (Figure S6 of Supporting Information). During the 6 h period, ~19% of the drug was released from the GO-IO-DOX at a pH of 7.4 while ~58% of the drug was released from the GO-IO-DOX at a pH of 5. This can be explained by the partial dissociation of hydrogen bond under acidic condition compared to the neutral pH. The hydrogen bonding interaction between DOX and GO is the strongest at the neutral condition and hence the minimum drug release was obtained at pH 7.4. Therefore we can confirm the π-π stacking interaction of DOX and GO, which contributed towards the continuous drug release at acidic pH. These results clearly indicate a pH-dependent drug release from GO-IO-DOX that is explained by the presence of hydrogen bonds between DOX and GO, which are more prominent in neutral conditions as mentioned earlier^21^. Therefore at a low pH, DOX becomes more hydrophilic and water-soluble, which thus leads to the release of more DOX from GO-IO-DOX into the aqueous solution^24^. As a result, this smart drug delivery platform can be considered to be unique in that it is inactive in normal tissue while the drug activity is triggered in cancerous tissue that has a low pH. Therefore the GO-IO-DOX has the potential to reduce the side effects caused by an early release of chemotherapeutic agents during circulation, thus improving the specific drug delivery.

**Figure 4:**
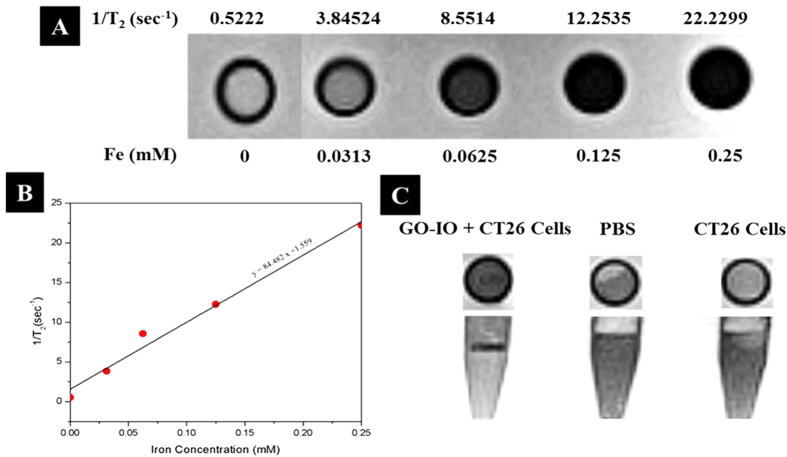
(**A**) T_2_ -weighted MR images of the GO-IO-PEG nanocomposites for various Fe concentrations, (**B**) Plot of the T_2_ relaxation rate (1/T _2_) against various iron concentrations of the GO-IO-PEG nanocomposites (relaxivity measurement of GO-IO-PEG nanocomposite by linear correlation between the relaxation rate 1/T_2_ and the iron concentration), (C) MR phantom imaging of CT26 cells with GO-IO-PEG nanocomposite.

The use of GO-IO-PEG nanocomposite as MRI contrast agents is verified by conducting T_2_-weighted MR imaging in a 3T clinical MRI instrument. The concentration-dependent darkening (T_2_ effects) of the GO-IO-PEG nanocomposite was measured (Fig. 4A), and the T_2_ contrast enhancement was observed to be directly dependent on the Fe concentration. The T_2_ relaxation rates (1/ T2) of GO-IO-PEG nanocomposites with various iron concentrations are plotted in Fig. 4B, and the transverse relaxivity (r_2_) of GO-IO was measured to be 84.0 L·mmol ^–1^ ·S ^–1^ which is far better than the IO nanoparticles alone (Figure S10 of Supporting Information). Thus, the results indicate that the GO-IO-PEG nanocomposites have great potential for use as MRI contrast agents at a very low concentration.

The MR sensitivity at the cellular level was investigated with a T_2_-weighted MRI of the CT26 cell lines incubated *in vitro* with GO-IO-PEG, while pure PBS and CT26 cell lines were kept as controls (Fig. 4C). The analysis of the T_2_ contrast enhancement effects indicated that the control cells did not produce a negative contrast relative to PBS while the treated cells showed an enhanced negative contrast effect as a result of the shortened T_2_ relaxation time due to the presence of superparamagnetic iron oxide nanoparticles (SPIONs) in the GO-IO nanocomposites taken up by the treated cells. Thus the GO-IO-PEG nanocomposite can be exploited as an efficient tool for MR-based cellular imaging due to its capability to achieve a high contrast and effective intracellular delivery.

**Figure 5:**
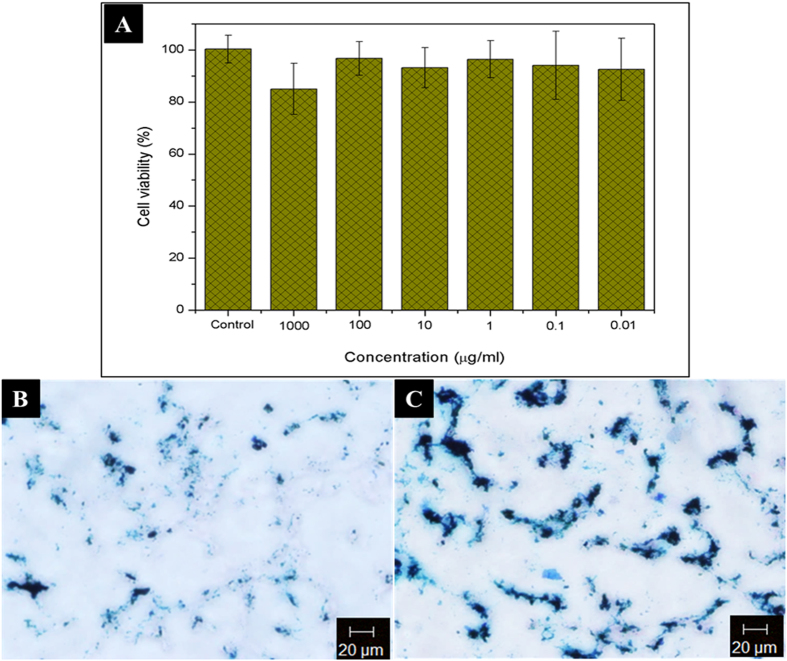
(**A**) *In vitro* biocompatibility of GO-IO-PEG nanocomposites at different concentrations, B,C Intracellular localization of GO-IO-PEG nanocomposites via Prussian blue staining in CT26 cell lines: (**B**) without a magnetic field and (**C**) with a magnetic field for 5 min.

Biocompatibility is an important characteristic that should be considered before a material is used for any biological application^34^,^35^,^36^. Fe_3_O_4_ nanoparticles are well known to be a promising candidate for use in biomedical applications because they possess excellent biocompatibility and stability in a physiological environment^37^. The cell viability of GO-IO-PEG samples was analysed after a 1 day period using an MTT assay in murine fibroblast (NIH3T3) cell lines. The cell viability was found to be almost equal to that of control for all concentrations ranging from 0.01 μg/ml to 1000 μg/ml, confirming its excellent biocompatibility (Fig. 5A).

The intracellular localization of nanoparticles (NPs) in tumors is very important for NP-assisted cancer diagnosis and treatment^38^. Based on the previous studies depicting an enhanced cellular uptake of magnetic nanocomposites under the application of a magnetic field^6^,^39^,^40^, the intracellular localization of GO-IO-PEG nanocomposites were evaluated in the presence of a magnetic field for 5 min prior to the incubation of nanocomposite with CT26 cell lines. For comparison samples were also incubated in the absence of a magnetic field. After 2 h of incubation, the intracellular uptake in both samples was evaluated by Prussian blue staining. The cell lines in both samples exhibited a very good uptake of the nanocomposites, even after a short incubation time of 2 hours (Fig. 5B), which can be explained by the good colloidal stability and smaller size of the nanocomposites^41^. Moreover, there was a substantial improvement in the intra cellular uptake of GO-IO-PEG nanocomposites for the same conditions in the AMF treated sample (Fig. 5C). This improvement in internalization may be a result of the increase in the cell membrane permeability without cell damage due to the application of mild AMF heating for a short period of time^6^.

**Figure 6:**
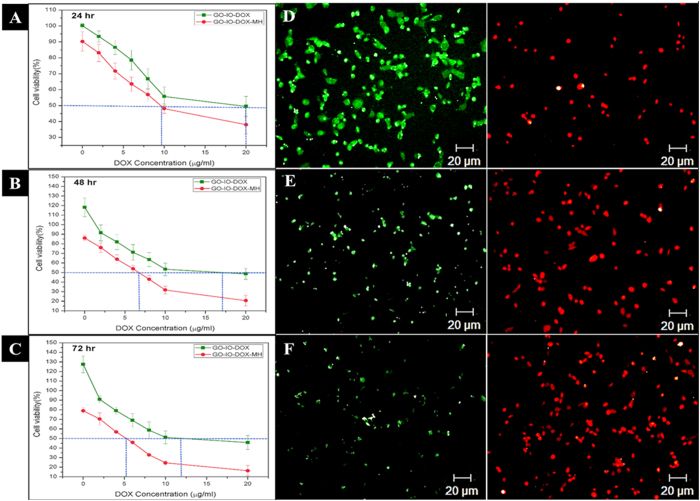
(**A–C**) Thermal enhancement of the drug cytotoxicity with periodic hyperthermia application in GO-IO-DOX, D-F Live/dead assay displaying the improved anticancer efficacy exhibited by the GO-IO-DOX due to the thermal enhancement of the drug cytotoxicity with a periodic application of hyperthermia.

Thermo-chemosensitisation refers to the thermal enhancement in drug cytotoxicity due to the concurrent hyperthermia application^10^. Several studies have reported the enhanced anticancer effect that can be achieved through combined therapy due to the thermo-chemosensitisation^42^. Here, the thermal enhancement of drug cytotoxicity was analysed by varying the DOX concentration from 0 to 20 μg/ml along with periodic hyperthermia (15 minutes/24 h) application for three cycles. As expected, the viability of CT26 cell lines was more suppressed by GO-IO-DOX–MH group compared to the GO-IO-DOX group with same drug concentration, explicitly depicting the enhanced drug cytotoxicity by the periodic application of magnetic hyperthermia (Fig. 6). Quantitatively, the drug concentration was analyzed by taking the half maximal inhibitory concentration (IC50)^43^ of the drug into consideration. The cytotoxicity of GO-IO-DOX toward the CT26 cell line was relative low in comparison to that of GO-IO-DOX-MH for the first 24 h, with a half-maximal inhibitory concentration (IC50) of ~20 μg while the GO-IO-DOX-MH group exhibited an IC50 of around ~9 μg (Fig. 6A). Moreover, the gap of the half maximal inhibitory concentration (IC50) between the GO-IO-DOX-MH (combined therapy) GO-IO-DOX (chemotherapy alone) groups significantly increased when the incubation time was extended from 48 to 72 h along with concurrent hyperthermia application (Fig. 6B,C). These results clearly indicate the thermal enhancement of DOX cytotoxicity due to the repeated application of mild hyperthermia. Therefore the prepared DOX loaded magnetic graphene nanocomposites significantly suppressed the cell viability at a very low DOX concentration when applied along with hyperthermia, thereby qualified as a novel material for cancer therapy with improved therapeutic efficiency with minimum side effects. A live/dead viability/cytotoxicity assay was conducted to qualitatively evaluate the thermal enhancement of the drug cytotoxicity. The drug concentration was fixed (12 μg/ml) and a periodic hyperthermia was also applied (Fig. 6D–F).

**Figure 7:**
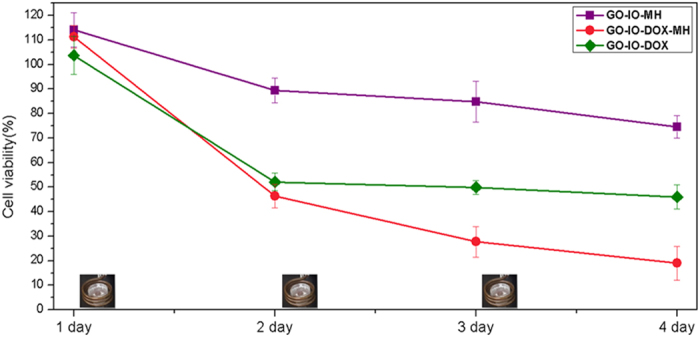
Effect of repeated hyperthermia on the anticancer efficacy of the CT26 cell line.

In order to compare the tumoricidal efficacy of the combination therapy (GO-IO-DOX-MH) with hyperthermia alone (GO-IO-MH) and chemotherapy alone (GO-IO-DOX), the anticancer efficacies were tested on the CT26 cell lines in the aforementioned three scenarios (Fig. 7). Periodic hyperthermia (15 min/24 h) was carried out on the GO-IO-DOX-MH groups and the GO-IO-MH groups while the GO-IO-DOX groups were kept as such. As expected, the GO-IO-DOX-MH group exhibited an increased cell cytotoxicity when compared to the other two samples, which clearly indicates a synergistic effect of the combined hyperthermia and chemotherapy. In the GO-IO-MH group, periodic hyperthermia for three cycles resulted in a moderate cell cytotoxicity of 26% due to the mild hyperthermia at a temperature of ~40 °C, whereas GO-IO-DOX-MH group exhibited an enhanced cell cytotoxicity of 82% due to both hyperthermia and drug activity. Therefore, the synergistic enhancement in the combined scenario can be explained to be a result of an increase in drug cytotoxicity due to the mild hyperthermia that was concurrently delivered. These results clearly indicate that the Graphene nanocomposite incorporated with magnetic nanoparticles as well as anticancer drug (GO-IO-DOX) has great potential to be used as an anticancer agent for cancer therapy.

**Figure 8:**
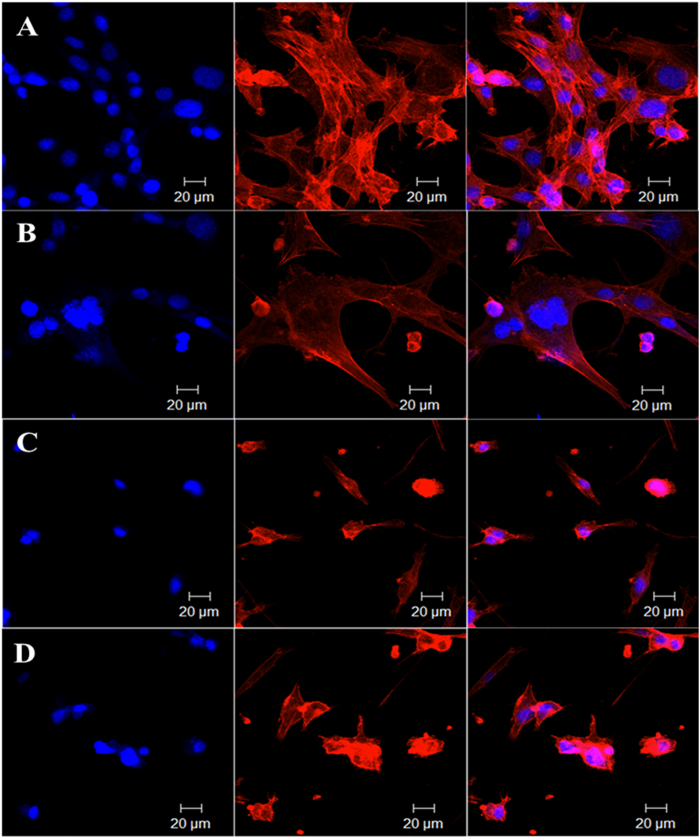
*In vitro* cytoskeletal imaging with Rhodamine B displaying localized tumoricidal effects of (**A**) GO-IO (control), (**B**) GO-IO-MH, (**C**) GO-IO-DOX, and (**D**) GO-IO-DOX-MH.

The localized tumoricidal effects of GO-IO-MH, GO-IO-DOX, and GO-IO-DOX-MH were qualitatively investigated by performing the cytoskeletal F-actin staining Rhodamine B (Fig. 8) on day 2 (24 h post for first hyperthermia application). The effect of GO-IO-MH (Fig. 8B), GO-IO-DOX (Fig. 8C), GO-IO-DOX-MH (Fig. 8D) on the F-actin organization in the cells can be clearly visualized by the change in cell morphology compared to control (Fig. 8A).

The typical feature of apoptosis such as membrane alteration and cytoskeletal damage including cell rounding and blabbing are clearly visualized in all the samples compared to control^10^. In the case of the GO-IO-DOX-MH group and GO-IO-DOX group, many of the cells exhibited shrunken morphology with membrane blebbing on their surface, whereas majority of the cells in GO-IO-MH group displayed good morphology. This indicates that GO-IO-MH group is responsible for a low cell death due to the application of mild hyperthermia only for 15 minutes, in contrast to GO-IO-DOX and GO-IO-DOX-MH. In GO-IO-DOX group, considerable cell death due to apoptosis has been taken place due to the activity of anticancer drug DOX; where as in GO-IO-DOX-MH group, apoptosis mediated enhanced cytotoxic effect was observed due to the thermal enhancement of DOX cytotoxicity. The results clearly indicate the enhanced efficacy of GO-IO-DOX-MH group for the synergistic anticancer therapy.

**Figure 9:**
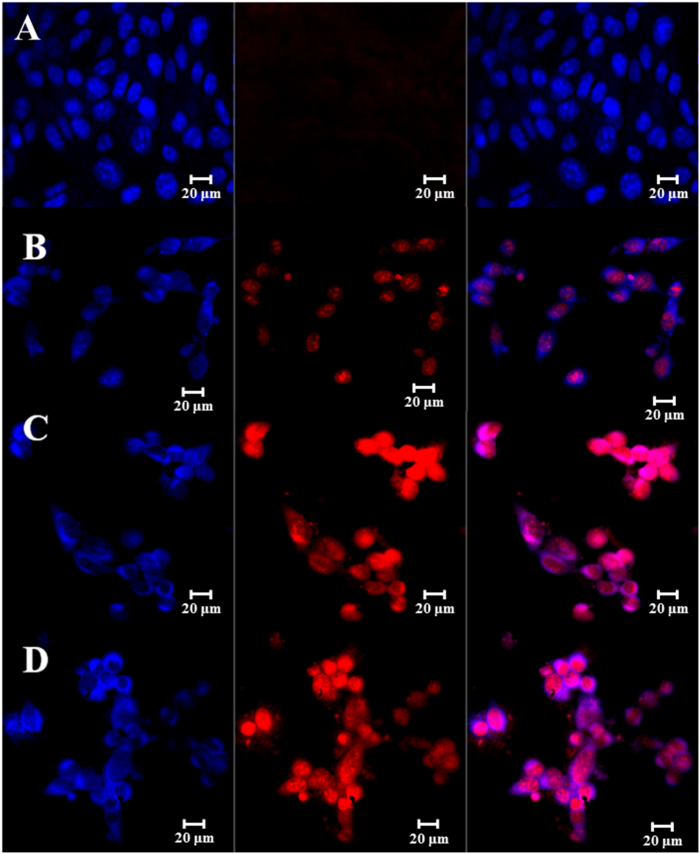
Magic Red^TM^ assay showing the apoptosis-inducing effect (red fluorescence) of GO-IO-DOX-MH due to drug sensitization through repeated hyperthermia, (**A**) Control GO-IO after 72 h of incubation, (**B**) GO-IO-DOX-MH 24 h post I MH, (**C**) GO-IO-DOX-MH 24 h post II MH (48 h) and (**D**) GO-IO-DOX-MH 24 h post III MH (72 h). The cell nuclei were stained with DAPI (blue fluorescence).

A Magic Red caspase detection kit was used to further evaluate the apoptosis-inducing effect of GO-IO-DOX-MH due to drug sensitization by repeated hyperthermia (Fig. 9). This test uses a red fluorogenic substrate for caspases 3 and 7, which are both known as essential proteases that play central roles in triggering apoptotic processes in mammalian cells^44^. In the GO-IO-DOX-MH group, the cells displayed a moderate red fluorescence throughout the cytoplasm in comparison with control for the 24 h post first cycle of hyperthermia application, suggesting that the GO-IO-DOX-MH considerably increased the caspase activation in the cancer cells (Fig. 9B) indicating a moderate cell death. Further two cycles of hyperthermia in the GO-IO-DOX-MH group resulted in a very high intensity of red fluorescence throughout the cytoplasm (Fig. 9C,D), suggesting the enhanced caspase activation in the cancer cells due to the increased cell death by apoptosis. This result indicates that the cell viabilities considerably decreased further due to the drug sensitization through repeated exposure to hyperthermia. Therefore we can conclude that GO-IO-DOX-MH significantly inhibited tumor growth by triggering apoptotic cell death.

## Conclusion

A multifunctional nanoplatform was successfully developed using Graphene Oxide, Iron Oxide and Doxorubicin (GO-IO-DOX). This platform is suitable for cancer theranostics because it provides multiple therapeutic functions in addition to imaging capabilities. The magnetic properties of these nanocomposites were tested, and a superparamagnetic behavior was observed with a very good magnetic field dependent heating ability and a cyclic heating profile. The T_2_ -weighted MR studies revealed the great potential of GO-IO nanocomposites as MRI contrast agents. The PEGylated GO-IO nanocomposites exhibited a very good biocompatibility with high cell uptake efficiency. The *in vitro* studies revealed that the GO-IO-DOX nanocomposites possess a greater therapeutic efficacy by exhibiting a superior hyperthermic response in an AMF and cancer cell specific DOX release to effectively exterminate the target cancer cells through apoptosis-mediated cell death. Furthermore the localized tumoricidal effects of the proposed GO-IO-DOX considerably increased due to the drug sensitization by repeated mild hyperthermia application. In a future study perspective, although the *in vitro* study results of GO-IO-DOX possess a great potential to be used as a cancer theranostics material, necessary *in vivo* studies has to be done to analyses the theranostics effect of thus developed GO-IO-DOX in a living model.

## Methods

### Materials

Commercial natural graphite powder (<150 micron), sodium Nitrate (NaNO_3_), KMnO_4_, conc. H_2_SO_4_, H_2_O_2_ iron (III) acetylacetonate [Fe(acac)_3_], 1,2-hexadecanediol (90%), oleic acid, oleylamine (70%), benzyl ether (98%), mPEG-NH_2_ (Mw 5000), N-Hydroxysuccinimide (NHS; 98%), Amine functionalized polyethylene glycol (mPEG-NH_2_; Mw 5000), N-(3-Dimethylaminopropyl)-N′-ethylcarbodiimide hydrochloride (EDC; ≥99.0%), Doxorubicin hydrochloride (98.0–102.0%), ethanol (99.9%), hexane (99.9%), chloroform ( 99.99%) were purchased from Sigma Aldrich, South Korea.

### Preparation of Graphene Oxide

Graphene oxide (GO) was synthesized using the modified Hummer’s method^26^. The detailed experimental procedure is as follows. 2 g of commercial natural graphite powder and 2 g of NaNO_3_ were added into 100 mL of concentrated H_2_SO_4_. The mixture was vigorously stirred in an ice bath for 30 min, and after full dispersion of the graphite in the acid, 7 g of KMnO_4_ was gradually added to the solution to oxidize the graphite. Stirring continued for another 1 h in an ice bath after the addition of KMnO_4_. Then, stirring continued overnight at room temperature to fully oxide the graphite. After the reaction was complete, 100 ml of distilled water (DI) were gradually added into the mixture to neutralize the reaction solution since a slow addition avoids unwanted explosions. After 30 min of stirring, the solution volume was increased to 500 ml by adding additional DI water. Then, 10 mL of 30% H_2_O_2_ solution were added to terminate the reaction and to enhance the oxidizing level. After the addition of H_2_O_2_ solution, the resulting solution was consecutively stirred, centrifuged, and then washed with 5% HCl until a neutral pH was reached. Graphene oxide was obtained after drying the centrifuged samples at 600 °C overnight in a hot air oven.

### Preparation of superparamagnetic iron oxide nanoparticles (SPIONs)

Monodisperse nanoparticles of size less than 20 nm were synthesized via classical thermal decomposition^25^. The amount of surfactants and solvent were adjusted in the reaction to control the size of the nanoparticles. In a typical synthesis procedure, 15 mmol of Fe(acac)_3_, 6 mmol of oleic acid, 6 mmol of oleylamine (surfactants), and 35 mL of benzyl ether (solvent) were mixed and magnetically stirred under a nitrogen flow, and the resulting mixture was heated to 165 °C under the protection of a nitrogen gas flow and was isothermally held for 30 minutes. The mixture was further heated to 280 °C and was maintained at this temperature for 30 min. The black-colored mixture that was thus obtained was allowed to cool down to room temperature, and the IONPs were precipitated by adding ethanol and were then separated by centrifugation. This washing procedure was repeated several times, and the IONPs were finally redispersed in chloroform at a concentration of 50 mg/ml and were kept in a sealed glass vial at 4 °C for further use.

### Preparation of amphiphilic graphene oxide Iron oxide nanocomposite (GO-IO)

The GO sheets were sonicated to truncate the size of GO to a size around 200 nm. The amphiphilic GO-IO nanocomposite was produced through a simple mini-emulsion method followed by a solvent evaporation process. Prior to carrying out the mini-emulsion method, the nanosized GO were functionalized with oleylamine. GO were grafted with oleylamine to facilitate the solubility of the GO in organic solvent and to initiate the hydrophobic–hydrophobic binding between GO and IONPs. Briefly, as-made GO (20 mg) were mixed with 7 mL of chloroform and 15 mmol of oleylamine, and the solution mixture was then sonicated with an ultrasonic homogenizer for 30 min. The unreacted oleylamine was washed by adding ethanol and was centrifuged at 10000 rpm for 10 min to separate the oleylamine-grafted nano GO. The precipitates were subsequently redispersed in chloroform at a concentration of 4 mg/ml with brief sonication. 1 mL of oleylamine grafted nano GO was subsequently mixed with 0.2 mL (50 mg/mL) of the nanoparticles dispersed in chloroform, and sonication was carried out for 5 min to obtain a uniform mixture. 12 mL of DI water were added by keeping the ratio of chloroform to water at 1:10, and the resulting mixture was then homogenized using an ultrasonic homogenizer for 30 minutes. The overall mixture was transferred to a pre-heated beaker at 70 °C, the organic solvents were allowed to evaporate, and impurities were further removed by centrifugation at 10 000 rpm for 10 min. The supernatant was collected and was stored at room temperature. The as prepared GO-IO nanocomposite was found to be very stable in organic solvents, such as hexane or chloroform, and exhibited partial solubility in water due to the presence of the inorganic functional group.

### Preparation of biocompatible and hydrophilic graphene oxide iron oxide nanocomposite (GO-IO-PEG)

The toxicity profiles of the graphene-based nanomaterials are not yet well understood, but several studies have confirmed the improved biocompatibility of functionalized GO with various biocompatible molecules, such as polyethylene glycol^45^, dextran^46^, gelatin^47^ and so on^17^ when compared to plain GO. Therefore the biocompatible GO-IO nanocomposite was prepared by grafting polyethylene glycol (PEG) functional groups on the GO-IO nanocomposite through the well-known EDC chemistry^48^.

Polyethylene glycol (PEG) functionalisation will improve the hydrophilicity, colloidal stability and biocompatibility of the GO-IO nanocomposites. To introduce PEG functionalisation, 9 mL of GO-IO nanocomposite (0.3 mg /mL) were mixed with 5 mL of mPEG-NH2 (40 mg/ mL) and were sonicated for 30 min. The reactive species was activated through the use of EDC-NHS chemistry: 3 mL of EDC (10 mg /mL) and 3 mL of NHS (8 mg/mL) were subsequently added to the mixture, stirred at 1000 rpm for 24 h, and then purified with DI water to obtain hydrophilic biocompatible GO-IO nanocomposites. Thus obtained hydrophilic biocompatible GO-IO nanocomposites were used to encapsulate the anticancer drug doxorubicin (DOX).

### Preparation of drug encapsulated GO-IO (GO-IO-DOX)

The anticancer drug doxorubicin was encapsulated in the PEGylated GO-IO nanocomposite by simple mixing to initiate physisorption. DOX loading was carried out by mixing different amounts of DOX with the GO–IO-PEG nanocomposite. A total of 10 mg of DOX were dispersed in 5 ml of DI water, and the dispersion was then sonicated for 10 min. Different concentrations of DOX (starting from 2 μg/ml) were then produced from stock (10 mg in 5 ml) in Eppendorf tubes with serial dilutions (all loadings were performed in triplicate), and these were sonicated for 2 min. A fixed amount of (100 μg/ mL) of GO-IO-PEG was added to each of the drug suspensions and was kept in a shaking incubator at room temperature. The drug loaded GO-IO-PEG (GO-IO-DOX) was separated from the free drug molecules via centrifugation at 12,000 r/min for 10 min. The drug loaded particles were then stored at 4 °C for further use. The concentration of the drug in the supernatant was measured from the UV spectra of DOX by using a calibration curve with a dilution series. The amount of drug that was incorporated into the GO-IO-PEG nanocomposites was estimated by subtracting the amount of supernatant with free drug from the total amount of drug that had been initially added.

### Material Characterizations

An X-Ray powder diffraction analysis was carried out on a Rigaku X-ray diffractometer (Cu Ka, k = 1.54059 Å) over Bragg angles ranging from 20° to 80° to characterize the crystal structures of GO, IONP and GO-IO. The size, morphology and crystallography of the samples were investigated via transmission electron microscopy (TEM, JEOL JEM, Japan), and the corresponding SAED (specific area electron diffraction) pattern was also studied. The chemical structures of GO and GO–IO were verified via FT-IR spectra using a Paragon 1000 Spectrometer (Perkin Elmer). Atomic force microscopy (AFM) images of GO-IO were obtained with a Nanoscope IV multimode, Digital Instrument Co., USA in the Tapping Mode. Micro-Raman spectroscopy (Nanofinder 30) with an argon ion laser at an excitation wavelength of 632.8 nm was used to confirm the different structural phases of the GO and IONP. The magnetic characterization of the samples was carried out on a physical property measurement system (PPMS, model 6000), and DOX binding and release were analyzed by capturing the UV–visible absorption spectra (HP 8453 UV–vis spectroscopy system, Germany) at a wavelength of 490 nm.

### *In vitro* Hyperthermia studies and SAR measurements

The heating induced by the alternating magnetic field (AMF) in the GO-IO-PEG nanocomposite was studied by placing an aqueous solution of GO-IO nanocomposites at various concentrations (50,100,1000 μg/ml) on the center of a water-cooled induction coil made of copper connected with an alternating magnetic field generator (OSH-120-B, OSUNG HITECH, Republic of Korea). The strength and frequency of the magnetic fields were adjusted to 12.57 kA/m and 293 kHz, respectively. The samples were heated for 900 s, and the heating characteristics were automatically recorded using type-T thermocouples and a real-time data acquisition system (NI-DAQ^R^, National Instruments, USA) with the Lab VIEW program. Before each experiment, the temperature was calibrated and stabilized for 10 min. The heating efficiency of the samples was then quantified by calculating the specific absorption rate (SAR), following the procedure that was earlier described^49^. The SAR values were then calculated using the equation (1) as.


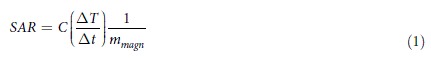


where C is the sample-specific heat capacity calculated as the mass-weighted mean value of the magnetite and water. The heat-capacity of the GO-IO is not considered in the current study since it is in a low concentration. Hence, the heat capacity of water (4.186 J g^−1^K^−1^) is considered as the heat capacity of the sample^50^. ΔT/Δt represents the initial slope of the time-dependent temperature curve, which was initially obtained for 60 s once after the magnetic field was switched on since the curve follows a linear relationship in this regime. The value for m_magn_ is considered to be the amount of magnetite per total amount of magnetite and water. The heating ability of the samples with repeated application of hyperthermia was also analyzed by using the same procedure where AMF is switched on and off.

### Kinetics of the pH-dependent drug release

The pH-dependent DOX release is analyzed from the GO-IO-DOX nanocomposites by making a suspension of GO-IO-DOX with a predetermined concentration of DOX in PBS buffer at physiological (pH 7.4) and acidic (pH 5) pH levels. Both samples were placed in a shaking incubator at 37 °C, and at different time intervals, 1 ml of the release solution (PBS) were taken out and replaced with 1 ml of fresh PBS to maintain a constant volume. The amount of DOX that was released was quantified by capturing the UV–visible absorption spectra at a wavelength of 490 nm. Triplicate samples were used to ensure the accuracy. The control graph has been already drawn using the known concentrations of drug at 490 nm. Using this control graph, the exact percentage of drug release from catecholic nanofibers was determined at different pH. The amount of drug incorporated into the GO-IO-DOX was estimated using the equation (2) as^51^:


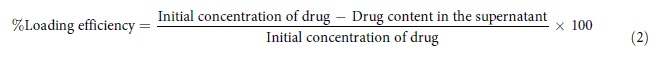


### Magnetic Resonance Relaxivity Analysis

T_2_-weighted MR imaging was carried out with various concentrations of Fe in the GO-IO-PEG nanocomposite. GO-IO-PEG was analyzed over 4 dilutions in a 1.5 ml centrifugation tube by using a 2-fold serial dilution starting from a concentration of 0.25 mM [Fe]. MRI experiments were carried out using a 3T clinical MRI instrument (Magnetom Tim Trio, Siemens Medical Solutions, Erlangen, Germany). The sagittal and axial images were obtained through a central section of the tubes, and the T2-weighted images were obtained using a gradient-echo sequence with TR = 1,0950, TE = 82, NEX = 4, slice thickness = 3 mm, flip angle = 180°, and FOV = 140 mm × 140 mm. The signal intensities of each region of interest (ROI) in the T_2_ map were measured for each concentration and were used to carry out specific relaxivity calculations. The relaxivity was calculated using a linear regression analysis (OriginPro8) of the relaxation rates and the molar Fe concentrations.

### *In vitro* cell culture studies

Murine fibroblast (NIH3T3), and murine colon carcinoma (CT26) cell lines were purchased from ATCC^®^ (Manassas, VA USA).

### *In vitro* MR Phantom Imaging

*In vitro* MR phantom imaging was carried out by evaluating the GO-IO-PEG taken up by the CT26 cell lines in phantom tubes. Initially 10^6^ CT26 cell lines were harvested from 6-well plate and were incubated with GO-IO-PEG at a concentration of 50 μg/ml for 2 hours. The cells were then washed with PBS and were fixed with 3.7% formaldehyde for 15 min. The cells were counted again and embedded in 10% gelatin (1:1) and were placed in 0.5 ml Eppendorf tubes in the middle gelatin layer for phantom MRI *in vitro*. PBS and the CT26 cell lines were kept as controls.

### In-Vitro cytocompatibility study

The cytocompatibility of the GO-IO-PEG nanocomposites against NIH3T3 cells was evaluated using the MTT assay. The cells were seeded onto a 96-well plate at a density of 10^4^ cells/well. The cells were cultured in a CO_2_ incubator at 37 °C in a humidified environment for one day, and the GO-IO-PEG was added to the cells in triplicate to analyze the cytocompatibility over the concentration range from 0.01 μg/ml to 1000 μg /ml. The cells were then incubated for 24 hours after treatment, and then, 20 μl of MTT reagent were added to each of the treated wells and were incubated for 4 hours. Finally, the absorbance at 490 nm was measured using a microplate reader.

### Intracellular localization study

The intracellular localization was qualitatively performed with Prussian blue staining, which is used to indirectly estimate the GO-IO uptake. The CT26 cell lines were treated with GO-IO-PEG with and without AMF for 5 min prior to incubation. Typically, CT26 cells (5 × 10^5^ cells/well) were seeded into an 8-well chamber slide (Lab-Tek2, Utah, USA) supplemented with medium (Thermo Scientific, Utah, USA). Both of the cell media contained 10% FBS and 1% penicillin–streptomycin, and the cells were kept overnight at 37 °C in a humidified 5% CO_2_ atmosphere. After 24 hours, the cells were washed with PBS twice to remove the medium. 100 μg/ml of GO-IO-PEG were added to the cells in duplicate, and a magnetic field was applied to one set for 5 minutes. Both samples were then incubated for another 2 hours to analyze the intracellular uptake. The cells were fixed with 4% PFA for 15 min and 100 μl of 4% potassium ferrocyanide (II) trihydrate and 4% HCl solution (in PBS) were added in each well. These were then incubated for another 20 minutes. The cells were counter-stained with nuclear fast red stain. The images were then collected on an inverted light microscope. The cells stained with blue color represent the iron oxide nanoparticle uptake and thereby the GO-IO uptake.

### *In vitro* hyperthermia induced chemo sensitization studies

The thermal enhancement of the drug cytotoxicity was first tested using GO-IO-DOX with DOX concentrations that varied from 0 μg/mL to 20 μg/mL with a GO-IO concentration fixed at 100 μg/mL. The cytotoxicity was then determined using an MTT assay. Briefly, CT26 cells were seeded at a density of 5 × 10^4^ cells into 24-well plates and were cultured in DMEM medium in an incubator with a humidified 5% CO_2_ atmosphere at 37 °C. After 24 h of incubation, 100 μg/ml of GO-IO-DOX with various concentrations of DOX was added to the cells, and the samples were incubated for 2h with an additional set of the same set of samples kept as controls. The samples were divided into two groups: one where hyperthermia was applied by switching on AMF (GO-IO-DOX-MH) and control groups where AMF was not applied (GO-IO-DOX). One set of plates (in triplicate) containing GO-IO-DOX-MH adherent cells was exposed to AMF for 15 min (12.57 kA/m, 293 kHz) in a sterile environment while the control samples (in triplicate) were isolated into a mini petri dish for the same period of time to cancel out environmental effects on cell death. After the hyperthermia treatment on all samples, fresh media was added and incubated in a 24-well plate again in an incubator with a humidified 5% CO_2_ environment at 37°. After 1 day, 50 μl of MTT reagent (Promega, USA) were added to each of the wells, and the plate was incubated for an additional 4 hours, and the absorbance at 490 nm was then measured using a microplate reader. The same experiment was repeated by applying periodic hyperthermia (15 min per 24 h) over consecutive days and the corresponding half maximal inhibitory concentration (IC 50) of the drug and the cell survival were assessed in further studies.

### *In vitro* hyperthermia study

Based on the half-maximal inhibitory concentration (IC50) of DOX in the control samples (GO-IO-DOX), the drug concentration was fixed to 12 μg/ml for use in further studies. The synergistic anticancer efficacy of the GO-IO-DOX-MH was tested in comparison to the hyperthermia (GO-IO-MH) and chemotherapeutic (GO-IO-DOX) effects alone. The cytotoxicity in the CT26 cell line was then evaluated by conducting an MTT assay, as previously explained, for 1, 2, 3 and 4 days.

### Qualitative analysis of localized tumoricidal effect

To assess the proportion of live and dead cells, a live/dead cell cytotoxicity assay (Molecular Probes, USA) were carried out. The treated cells (24 h post hyperthermia) were stained with 200 μl of calcein AM (4 μM) and ethidium homodimer (0.5 μM) dissolved in PBS for 20 minutes at room temperature. The coverslip containing the cells was viewed directly under a fluorescent microscope with a standard fluorescein band pass filter for calcein and a Texas Red^®^ dye filter for ethidium homodimer. The same procedure was then repeated for the hyperthermia treatment on 48 and 72 h. The assessment of cell cytotoxicity and cytoskeletal damage were qualitatively studied by performing the cytoskeletal F-actin staining Rhodamine B (Molecular probes, USA). The cell nuclei were stained with DAPI (4,6-diamidino-2-phenylindole, dilactate, Invitrogen, USA). The apoptosis-inducing effect of GO-IO-DOX resulting from the drug sensitization due to repeated hyperthermia was qualitatively evaluated by using a Magic Red caspase detection kit that uses a red fluorogenic substrate for caspases 3 and 7. The treated cells (24 h post hyperthermia), were further incubated with Magic Red caspase detection kit for 45 mins. The cell nuclei were stained with DAPI. The same procedure was then repeated for the hyperthermia treatments on 48 and 72 h. The cells were observed using a LSM510 confocal laser scanning microscope.

## Additional Information

**How to cite this article**: Ramachandra Kurup Sasikala, A. *et al*. Multifunctional Nanocarpets for Cancer Theranostics: Remotely Controlled Graphene Nanoheaters for Thermo-Chemosensitisation and Magnetic Resonance Imaging. *Sci. Rep.* **6**, 20543; doi: 10.1038/srep20543 (2016).

## Supplementary Material

Supplementary Information

## Figures and Tables

**Figure 1:**
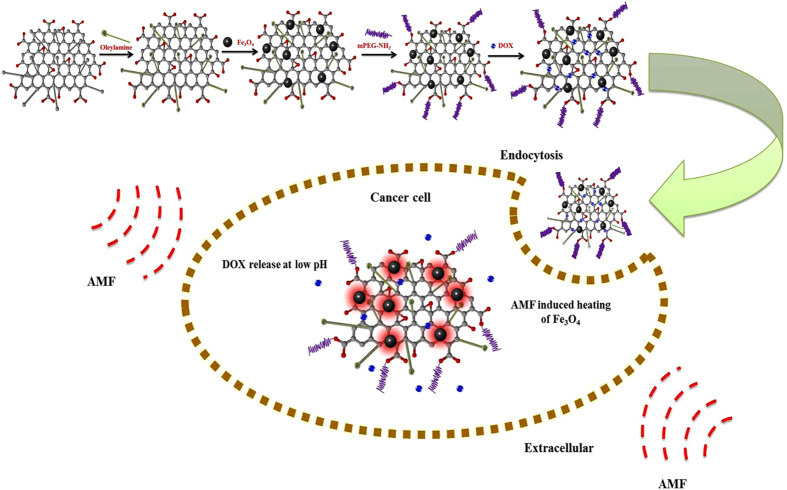
Schematic illustration showing the overall concept of the present study.
