# Supplementary material for: Multifunctional Nanocarpets for Cancer Theranostics: Remotely Controlled Graphene Nanoheaters for Thermo-Chemosensitisation and Magnetic Resonance Imaging
Source: Sci Rep. 2016 Feb 4;6:20543. doi: 10.1038/srep20543 (PMC4740792; doi:10.1038/srep20543)
Supplement: Supplementary Information [file srep20543-s1.doc]

**Supporting Information**

**Multifunctional Nanocarpets for Cancer Theranostics: Remotely Controlled Graphene Nanoheaters for Thermo-Chemosensitisation and Magnetic Resonance Imaging**

**Arathyram Ramachandra Kurup Sasikala ^1‡^, Reju George Thomas^3‡^, Afeesh Rajan Unnithan^1, 2*^, Balasubramaniam Saravanakumar, Yong Yeon Jeong^3^, Chan Hee Park^2*^ and Cheol Sang Kim ^1, 2*^**

*^1^Department of Bionanosystem Engineering, Graduate School, Chonbuk National University, Jeonju 561-756, Republic of Korea*

*^2^Division of Mechanical Design Engineering, Chonbuk National University, Jeonju 561-756, Republic of Korea*

*^3^Department of Radiology, Chonnam National University Hwasun Hospital, Chonnam National University Medical School, Gwangju 501-746, Republic of Korea*

((^‡^ both authors contributed equally to the work))

E-mail: ([chskim@jbnu.ac.kr](mailto:chskim@jbnu.ac.kr) , [biochan@jbnu.ac.kr](mailto:biochan@jbnu.ac.kr), afeeshnano@gmail.com )

**Schematic diagram illustrating the procedure followed in the preparation of GO-IO-DOX**


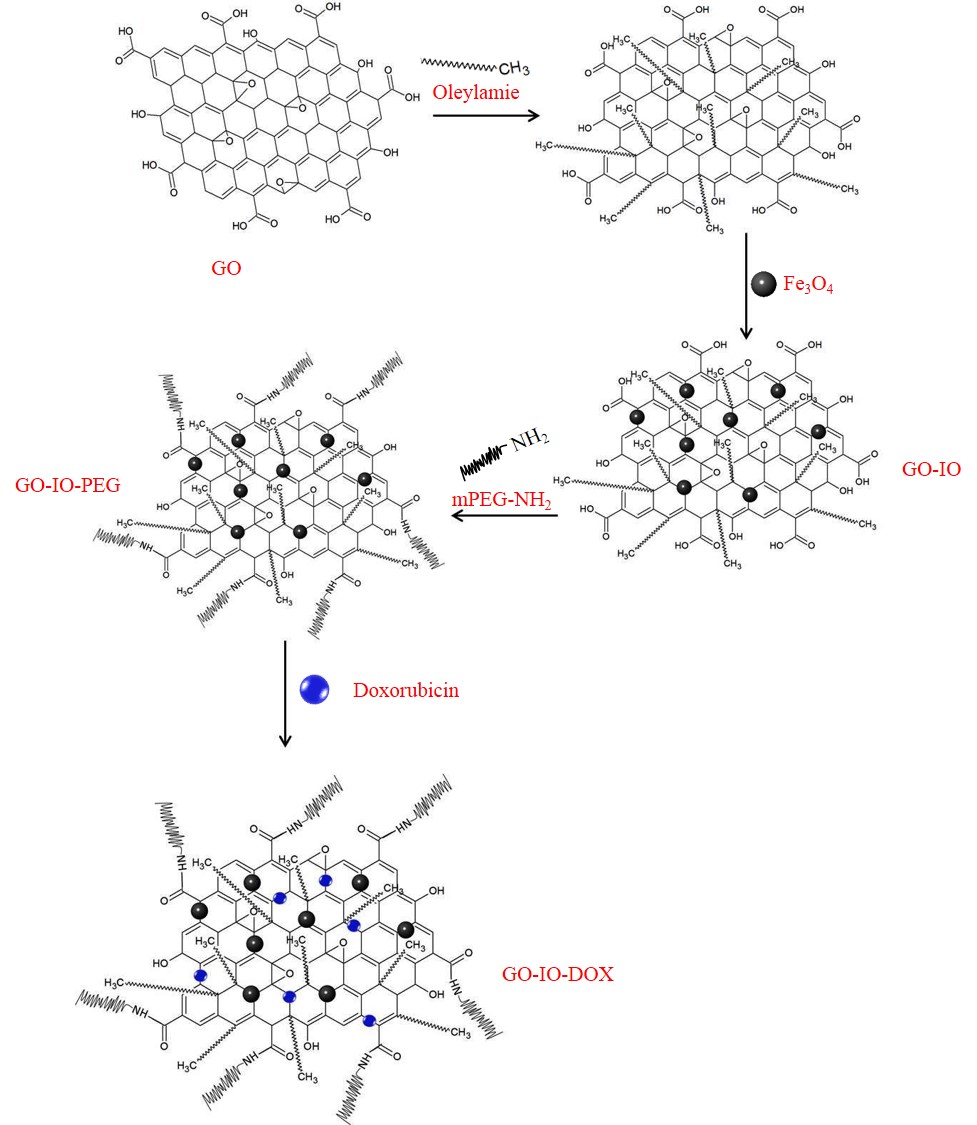


**Figure S1:** Schematic diagram illustrating the procedure followed in the preparation of GO-IO-DOX

**Morphology analysis using HRTEM**

Figure. S2 shows the TEM images of the graphene oxide and the monodisperse IONPs. The TEM image of the GO clearly exhibits the typical wrinkle morphology of GO with a single or very thin layer (Figure S2A). The well-defined diffraction spots in SAED (Figure S2C) illustrate the crystalline state of the GO. Figure S2B shows an HRTEM image of IONPs (Fe_3_O_4_) with corresponding SAED pattern (Figure S2D). The TEM image reveals that the nanoparticles possess a uniform distribution with a spherical morphology. The IONP prepared via thermal decomposition had sizes of less than 15 nm, and the ring-like SAED patterns indicate that the particles had a polycrystalline structure ^1^.


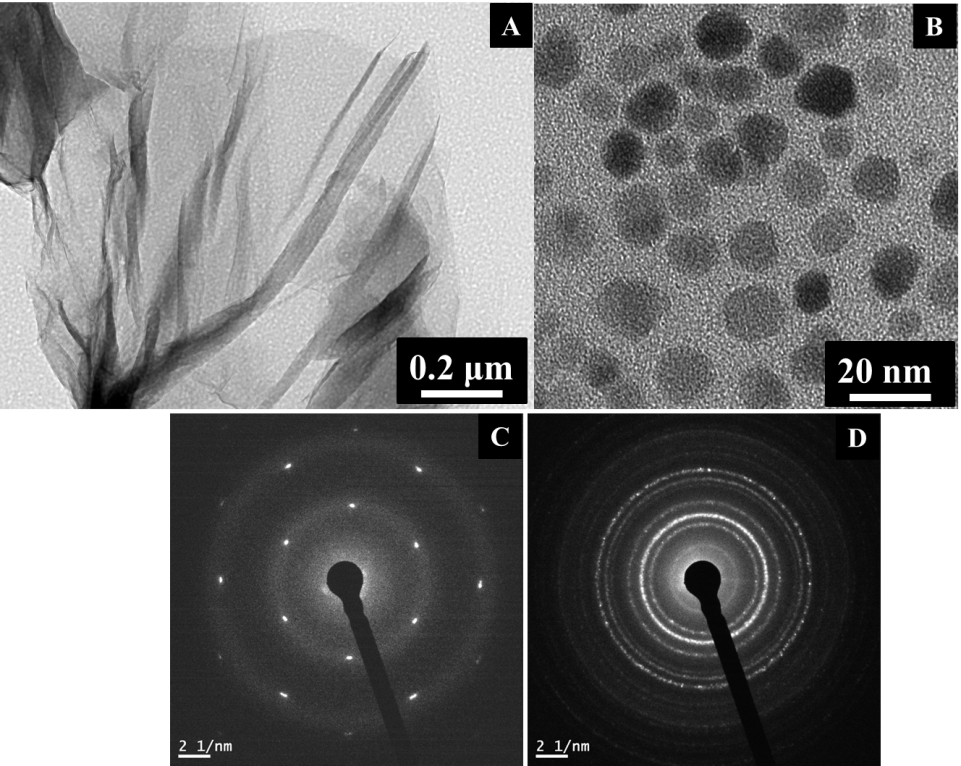


**Figure S2:** HRTEM and the corresponding SAED images of the as prepared A&C) GO and B&D) IONPs.

**Structural Characterization using XRD**

The XRD patterns of the GO and GO–IO nanocomposites are presented in Figure S3. GO shows a sharp peak centered at 2θ = 10.6°, which corresponds to an (002) inter-planar spacing of 0.84 nm. The diffraction peaks that appeared at 2θ=18.2° (111), 30.1° (220), 35.5° (311), 43.1° (400), 53.4° (422), 56.9° (511), 62.5° (440) and 73.9° (533) are characteristic of Fe_3_O_4_ crystals with a cubic spinel structure (JCPDS 19-062). When compared to Fe_3_O_4_ particles, the as-prepared GO–Fe_3_O_4_ composite materials present all diffraction peaks of the Fe_3_O_4_ combined with GO. In addition, there were no apparent shifts in the diffraction peaks. These results indicate that the Fe_3_O_4_ nanoparticles were well loaded on the GO sheets and that the Fe_3_O_4_ nanoparticle loading does not affect the GO structure during all experimental processes ^2^.


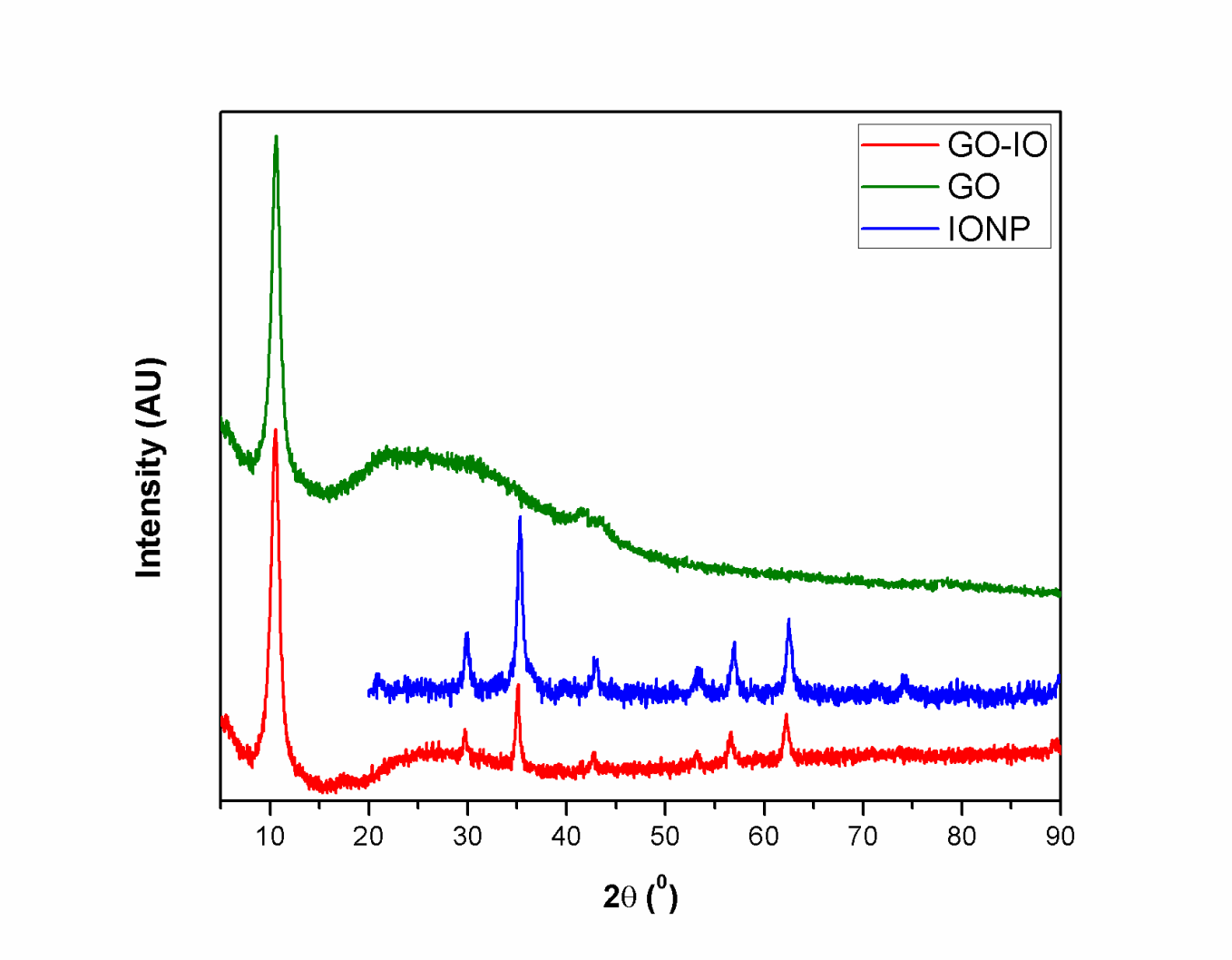


**Figure S3:** XRD patterns of the GO, IONPs and GO-IO nanocomposites.

**Raman spectroscopy Analysis**

Raman spectroscopy is powerful, non-destructive tool that can be used characterize carbonaceous materials, particularly to distinguish the ordered and disordered crystal structures of carbon. Figure S4. shows the Raman spectra of the GO and GO–IO nanocomposite. The Raman spectrum of GO shows two strong peaks at 1335 cm^-1^ and 1598 cm^-1^ that correspond to the D band and G band, respectively. The D band is usually an indication of disordered GO originating from defects associated with vacancies, grain boundaries and amorphous carbon species while the G band indicates the presence of the E_2g_ phonon of C sp^2^ atoms in a 2-dimensional hexagonal lattice. The Raman spectrum of the GO–IO nanocomposite shows typical magnetite peaks, and the characteristic GO peaks at around 1324 cm^-1^ and 1592 cm^-1^ correspond to the D and G bands, respectively ^3^.


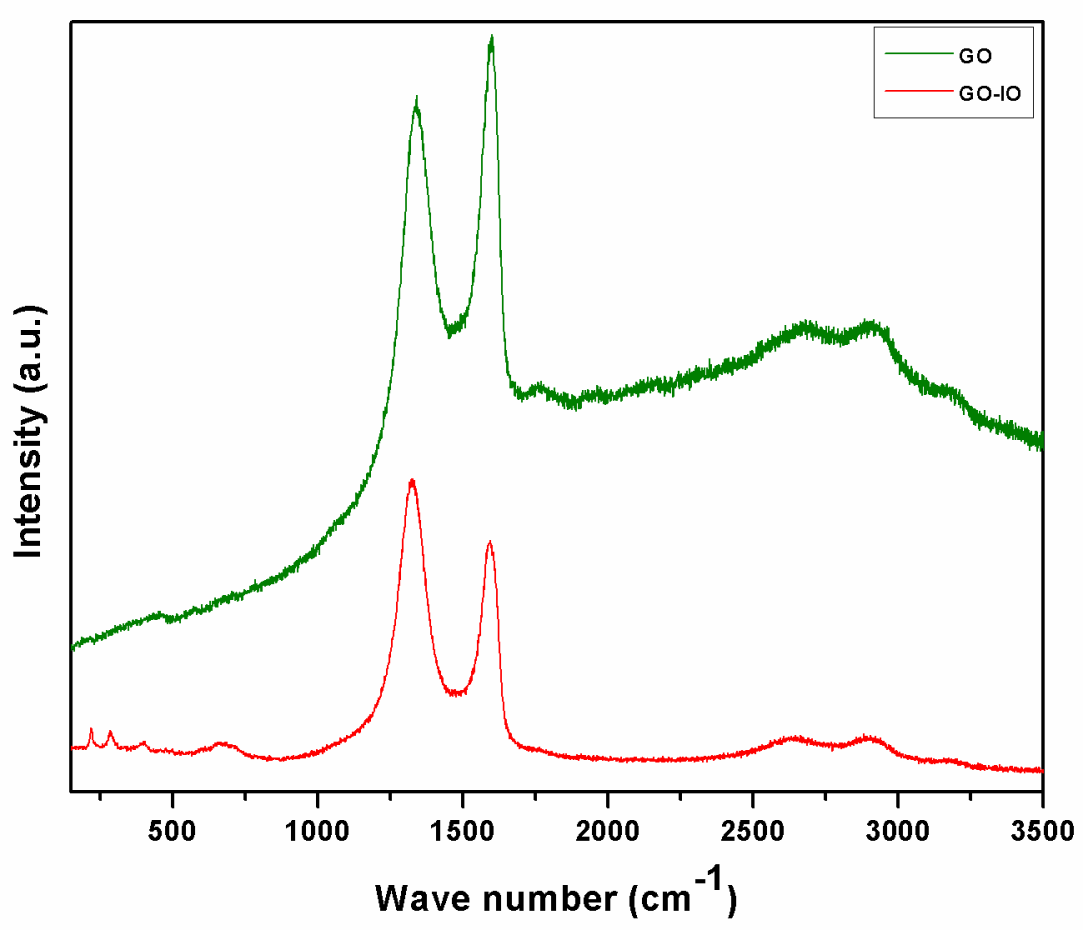


**Figure S4:** Resonance Raman spectra of GO and GO-IO nanocomposites

**Thermal gravimetric analysis (TGA)**

TGA experiments were carried out to determine the fraction of the magnetic nanoparticles in the GO-IO nanocomposites. The thermal behavior of the GO-IO nanocomposites exhibited ~70 % weight loss (Figure S5). This weight losses was a result of the removal of organic moieties, and therefore, remaining ~30% was attributed to the magnetic components of the GO-IO nanocomposites.


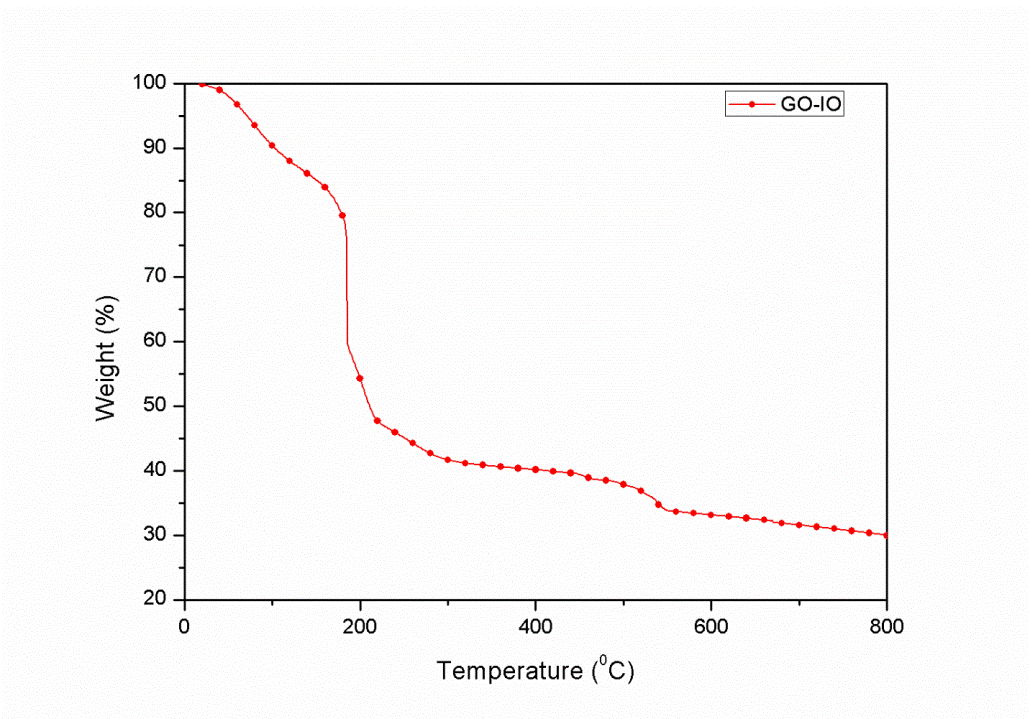


**Figure S5:** TGA of GO-IO nanocomposite


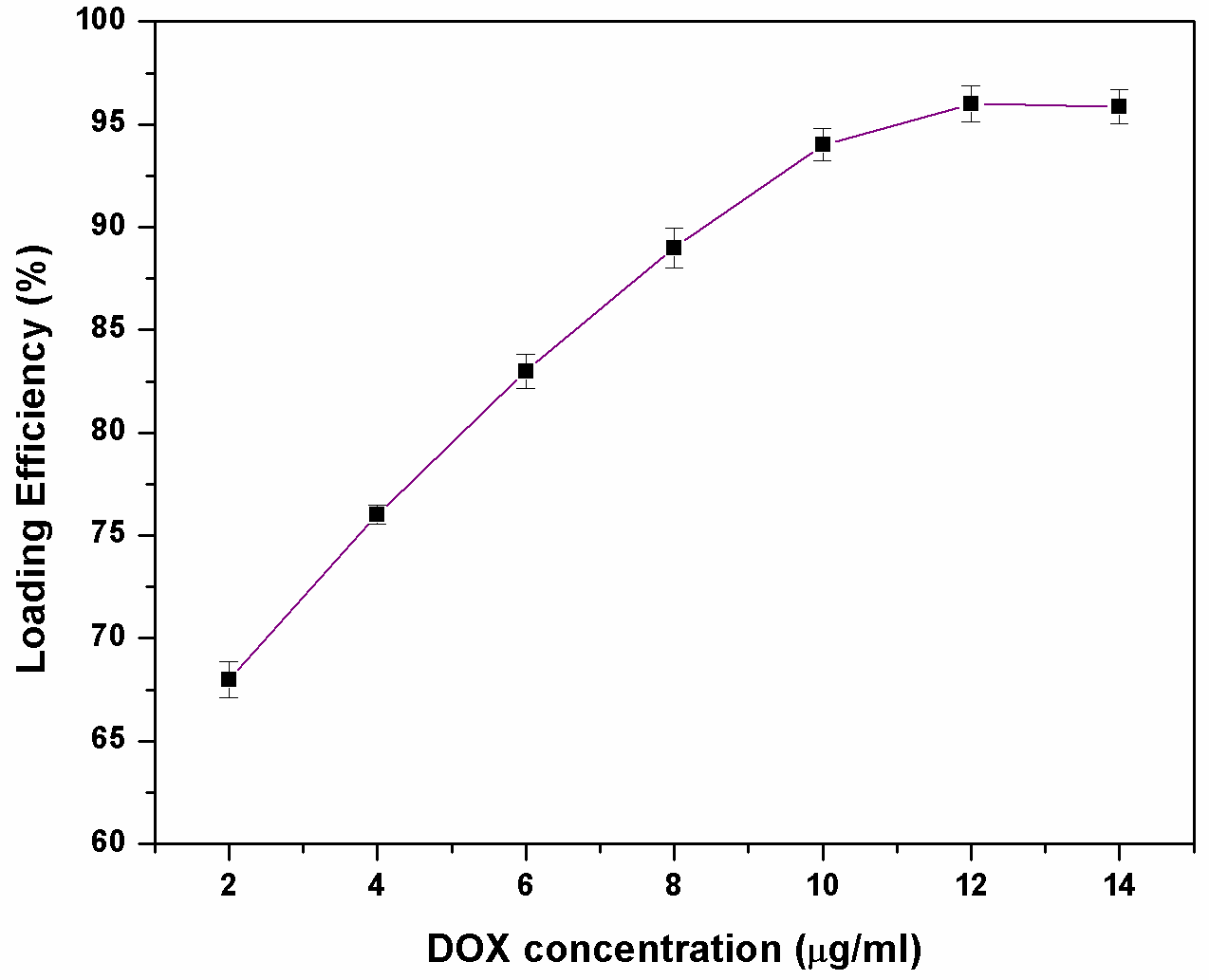


**Figure S6:** Loading efficiency of DOX in GO-IO composite (100μg/ml)


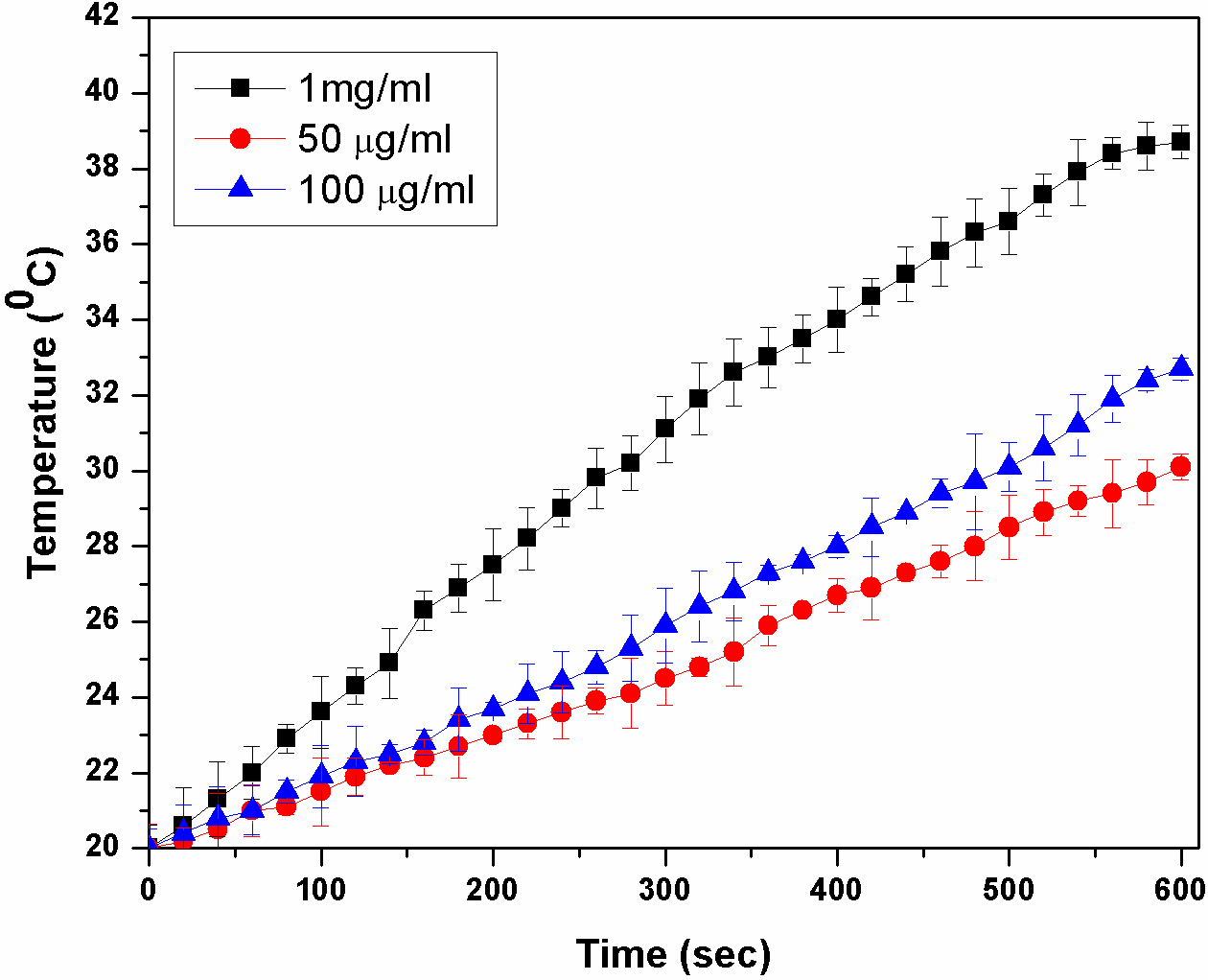


**Figure S7:** Magnetic field dependent heating ability of IO nanoparticles at various concentrations

**UV-VIS photospectroscopy of GO-IO and GO–IO–DOX**

The DOX loading in the GO–IO–DOX nanocomposites was evaluated using UV-VIS photospectroscopy. The characteristic UV–vis absorbance peak of DOX at 490 nm can be observed to be superimposed on the GO–IO absorption spectrum (Figure S7). As expected, a significant DOX fluorescence quenching effect was observed in the GO–IO–DOX sample at 490 nm due to the close binding between the DOX molecules and the graphene surface.


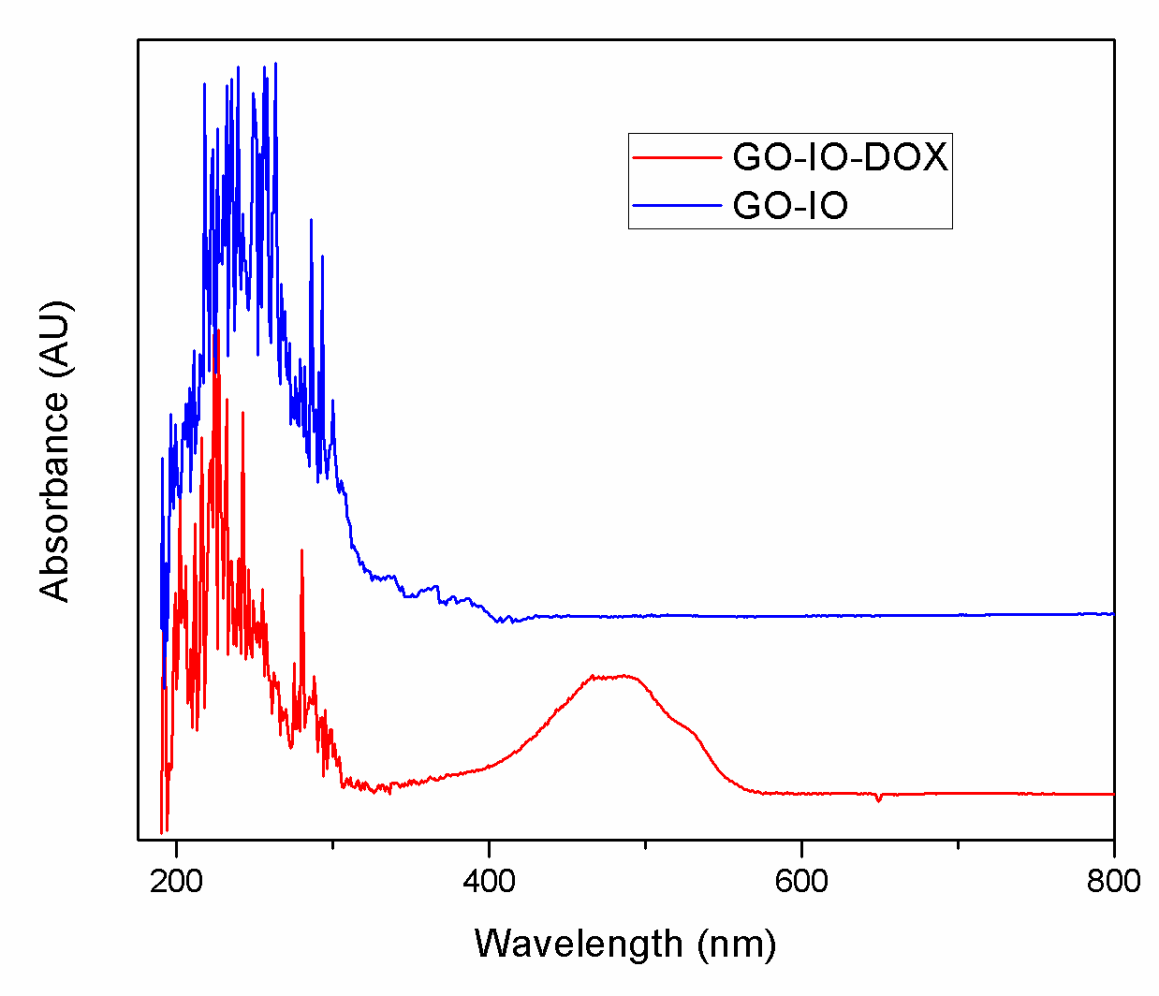


**Figure S8:** A) UV–vis absorbance spectra of GO-IO and GO–IO–DOX

**pH dependent release of DOX from GO–IO–DOX**

The pH-sensitive release of DOX from GO-IO-DOX was analyzed by testing the release of DOX in buffer solutions at a pH of 5 and 7.4 to mimic the pH of a tumor environment and of normal tissue or blood, respectively, and drug release was found to have been triggered in the acidic pH (Figure S8). The DOX released from GO–IO–DOX was measured with an UV-visible spectrophotometer at 490 nm. The control graph was already drawn using known concentrations of the drug at 490 nm, and this control graph was used to determine the exact percentage of drug that was released from the nanocomposite at a different pH. The results indicate that GO–IO–DOX exhibits a pH-dependent drug release that is triggered at low pH.


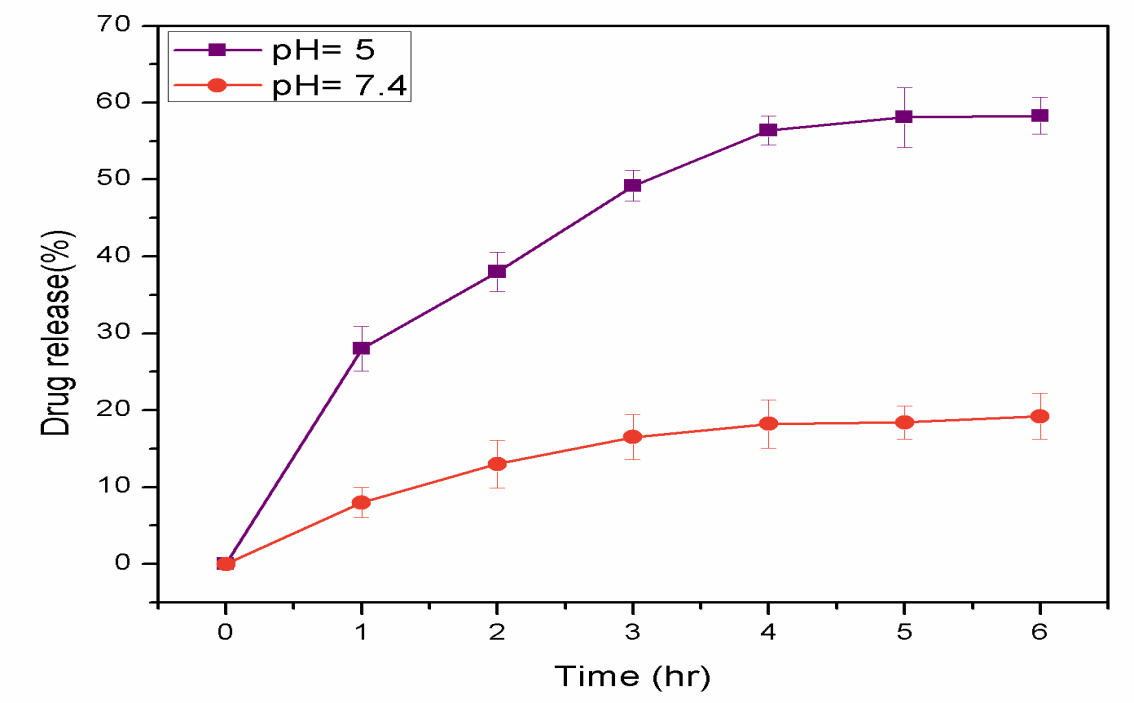


**Figure S9:** pH dependent release of DOX from GO–IO–DOX over time


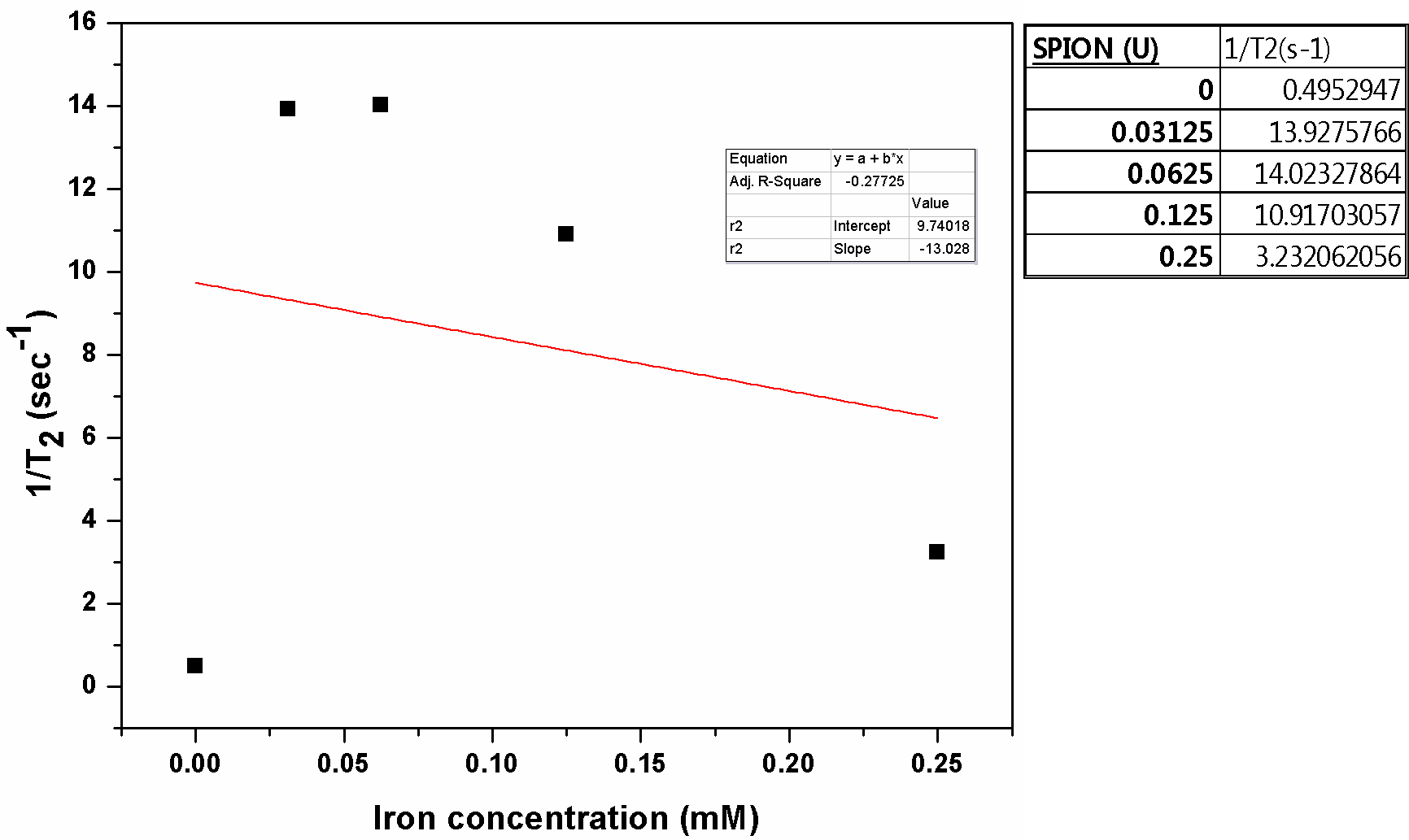


**Figure S10:** Plot of the T_2_ relaxation rate (1/ T _2_) against various iron concentrations of the IO nanoparticles (r2=-13.0280 L·mmol ^–1^ ·S ^–1^)

**References**

1. 1 Guo, Z. C. *et al.* Dandelion-like Fe3O4@CuTNPc hierarchical nanostructures as a magnetically separable visible-light photocatalyst. *J Mater Chem* **21**, 12083-12088, doi:DOI 10.1039/c1jm11098e (2011).
2. 2 Liang, J. J. *et al.* Flexible, Magnetic, and Electrically Conductive Graphene/Fe3O4 Paper and Its Application For Magnetic-Controlled Switches. *J Phys Chem C* **114**, 17465-17471, doi:DOI 10.1021/jp105629r (2010).
3. 3 Xie, G. Q. *et al.* A facile chemical method to produce superparamagnetic graphene oxide-Fe3O4 hybrid composite and its application in the removal of dyes from aqueous solution (vol 22, pg 1033, 2012). *J Mater Chem* **22**, 25485-25485 (2012).
